# Contact application of neonicotinoids suppresses the predation rate in different densities of prey and induces paralysis of common farmland spiders

**DOI:** 10.1038/s41598-019-42258-y

**Published:** 2019-04-05

**Authors:** Milan Řezáč, Veronika Řezáčová, Petr Heneberg

**Affiliations:** 1 Crop Research Institute, Biodiversity Lab, Prague, Czech Republic; 2 Czech Academy of Sciences, Institute of Microbiology, Prague, Czech Republic; 3 Charles University, Third Faculty of Medicine, Prague, Czech Republic

## Abstract

Neonicotinoids are very effective in controlling crop pests but have adverse effects on predators and pollinators. Spiders are less sensitive to neonicotinoids compared to insects because of the different structure of their acetylcholine receptors, the binding targets of neonicotinoids. We tested whether short-term exposure to neonicotinoids affected the predation rate in different densities of prey of spiders and led to their paralysis or eventual death. To examine these effects, we topically exposed dominant epigeic, epiphytic and sheet-weaving farmland spiders to four widely used neonicotinoids (imidacloprid, thiamethoxam, acetamiprid and thiacloprid). We applied the neonicotinoids at concentrations recommended by the manufacturers for spray application under field conditions. Short-term exposure to the formulations of all four tested neonicotinoids had adverse effects on the predation rate of spiders, with imidacloprid (Confidor) associated with the most severe effects on the predation rate and exhibiting partial acute lethality after one hour (15–32%). Acetamiprid also displayed strong sublethal effects, particularly when applied dorsally to *Philodromus cespitum*. Day-long exposure to dorsally applied acetamiprid or thiacloprid led to paralysis or death of multiple Linyphiidae spp., with the effects particularly prominent in males. To conclude, we provided multiple lines of evidence that short-term exposure to neonicotinoids, which were applied at recommended field concentrations, caused severe health effects or death in multiple families of spiders. Even acetamiprid caused strong effects, despite being subject to less strict regulations in the European Union, compared with those for imidacloprid because of claims of its negligible off-target toxicity.

## Introduction

Neonicotinoids are very effective in controlling crop pests, such as aphids, but have adverse effects on predators^1–3^ and pollinators^4–7^. Despite various predators and pollinators attracted the most attention concerning the examination of pesticides’ effects on beneficial arthropods^8^ and despite spiders are among the economically important predators, the data on sublethal effects of neonicotinoids on spiders are insufficient and conflicting. Spiders provide important ecosystem services in agricultural landscapes. For example, spiders reduce populations of the codling moth, *Cydia pomonella* (Lepidoptera: Tortricidae), and pear psyllid, *Psylla pyri* (Hemiptera: Psyllidae) in orchards^9,10^. The mortality of spiders in response to exposure to neonicotinoids is lower than that of multiple groups of insects^11,12^. The lower toxicity to spiders is likely because the structure of acetylcholine receptors, which mediate the action of neonicotinoids in arthropods, differs between insects and spiders. In spiders, acetylcholine receptors are present^13,14^ but their sensitivity to neonicotinoids is lower than that of insect acetylcholine receptors^15^. Although the lethality of neonicotinoids is limited with regards to spiders, the evidence for sublethal effects of neonicotinoids to spiders is conflicting. Widiarta *et al*.^16^ and Uhl *et al*.^17^ documented decreased prey consumption by *Pardosa pseudoannulata* (Araneae: Lycosidae) and *Pisaura mirabilis* (Araneae: Pisauridae), respectively, when subjected to imidacloprid exposure. By contrast, Řezáč *et al*.^18^ found that exposure to acetamiprid did not cause changes in the predatory ability of the spider *Philodromus cespitum* (Araneae: Philodromidae) to prey.

The use of neonicotinoids has rapidly increased worldwide^19,20^, with only very recent attempts to ban their use in the European Union^21,22^. The decline in insectivorous birds linked to neonicotinoids^23^ indicates that the off-target and indirect effects are likely broader than what the currently available direct evidence suggests^24–26^. Commercially distributed neonicotinoids are water-soluble and break down relatively slowly in soil/sediment matrices, with the half-life spanning from several weeks when exposed to sunlight to nearly four years in the absence of sunlight and the activity of microorganisms. Conclusive evidence demonstrated their accumulation in soil and irrigation channels for up to two years after treatment^27,28^. They are also found in the foliage of off-target plants growing in field margins^29^. Thus, all farmland plant-dwelling or soil-dwelling arthropods, including their predators and parasitoids, may come in contact with neonicotinoids. The extent of neonicotinoids’ effects remains incompletely understood. Their effects may not only include the obvious lethal effect but several sublethal effects have also been reported^8,30^. These include changes in foraging behavior, memory and learning, which all were reported from extensive studies of honeybees^31–33^. Transgenerational hormesis, measured as the effects of exposure of a parental generation on the reproduction and longevity of its progeny, was reported in *Aphis gossypii* and *Aphis glycines* (Hemiptera: Aphididae)^34,35^. Concerning the seed-applied neonicotinoids, recent meta-analysis by Douglas and Tooker revealed that the neonicotinoids reduced the abundance of arthropod natural enemies to the similar extent as applications of pyrethroid insecticides^12^. Different modes of exposure may play a role as shown, for example, in the study of thiamethoxam effects on *Serangium japonicum* (Coleoptera: Coccinellidae), an important predator of *Bemisia tabaci* (Hemiptera: Aleyrodidae), which responded most strongly to the systemic exposure, followed by egg-dip, and was only slightly affected by a dry residue exposure^36^. The toxicity of neonicotinoids can also have delayed and time-cumulative effects as reported for multiple insect taxa^37,38^.

Based on the previous conflicting evidence, we hypothesized that neonicotinoids have negative effects on the predation rate in different densities of prey of farmland spiders and may lead to spider paralysis or eventual death. Thus, we topically exposed dominant epigeic (*Pardosa lugubris* (Walckenaer, 1802) (Araneae: Lycosidae)), epiphytic (*Philodromus cespitum* (Walckenaer, 1802)) and sheet-weaving (multiple Araneae: Linyphiidae spp.) spiders of central European farmlands to four neonicotinoids that are widely sprayed in foliar applications in agriculture (imidacloprid, thiamethoxam, acetamiprid and thiacloprid) and analyzed changes in predation rate in different densities of prey, induction of paralysis and mortality.

## Results

### Mortality of epigeic and epiphytic spiders

**Figure 1:**
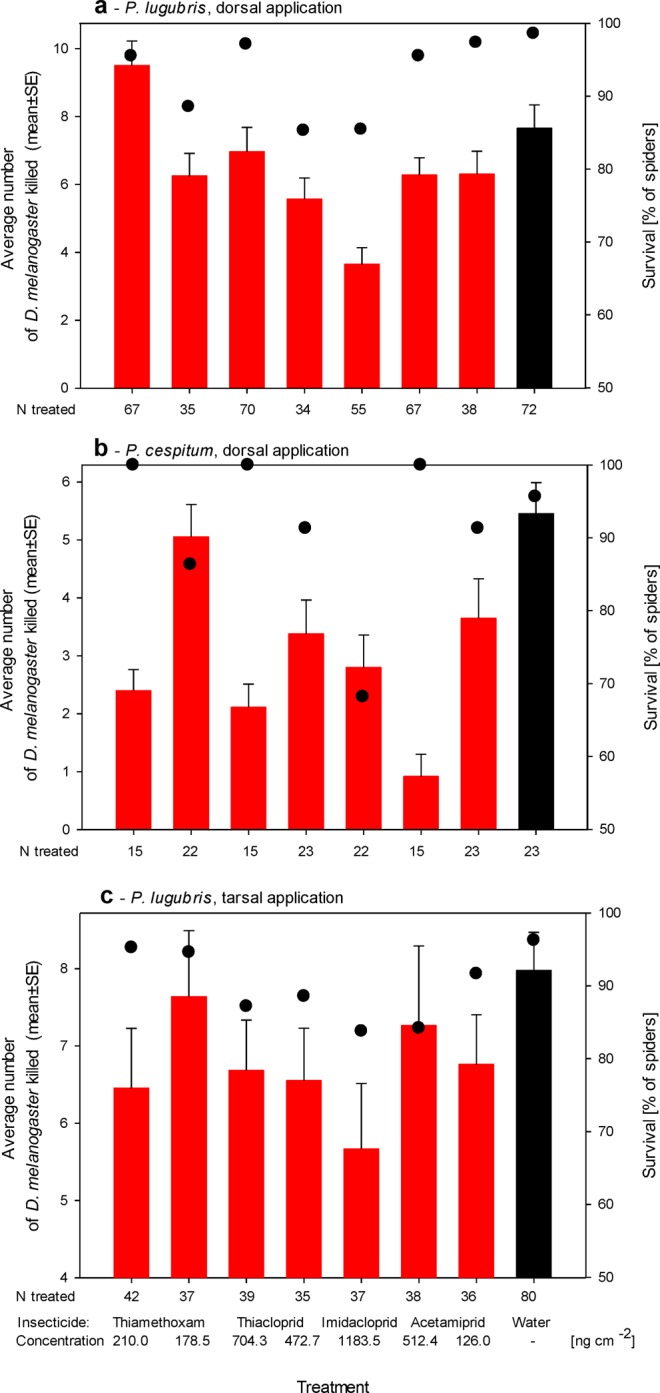
The effects of neonicotinoids on the predation rate in different densities of prey and survival of common orchard spiders. Columns show the mean predation rate in different densities of prey of spiders in the three experiments. The predation rate is expressed as the number of *D. melanogaster* killed during the experiment; whiskers indicate the SE of the means. Dots show the survival of spiders during the initial 1-hour-long exposure to the study compounds. The applied treatments are indicated on the X-axes. Red columns indicate cohorts that were treated with the indicated insecticide; black columns indicate cohorts treated with distilled water only.

Short-term exposure to the neonicotinoid imidacloprid at 1183.5 ng cm^−2^ was lethal to 15–32% of the spiders (Fig. 1). Imidacloprid caused a significant decrease in survival when applied to both tested spider species and using both modes of exposure with the spiders (*P. lugubris*, dorsal application, survival 85%, χ^2^ = 6.321, *D*_*f*_ = 1, *P* = 0.01; *P. cespitum*, dorsal application, survival 68%, Fisher’s exact test *P* = 0.02; *P. lugubris*, tarsal exposure, survival 84%, χ^2^ = 3.921, *D*_*f*_ = 1, *P* < 0.05). Trends toward decreased survival were also observed following exposure to the other neonicotinoids. However, the post-test power analysis revealed that the experiments were not sufficiently powered to test for the observed mild differences. Only two applications reached a power of >45% at α = 0.05. These were represented by the tarsal exposure of acetamiprid to *P. lugubris* at 512.4 ng cm^−2^ (84% survival) and the dorsal application of thiacloprid to *P. lugubris* at 472.7 ng cm^−2^ (85% survival). In both of these groups that passed the power test threshold, the survival was significantly lower than that in the respective control groups (χ^2^ = 3.729, *D*_*f*_ = 1, *P* = 0.05 and χ^2^ = 5.379, *D*_*f*_ = 1, *P* = 0.02, respectively).

### Predation rate in different densities of prey of epigeic and epiphytic spiders

Predation rate in different densities of prey of the tested spiders, measured as the predatory ability to catch *D. melanogaster* flies, decreased significantly following the dorsal application of neonicotinoids; whereas we did not observe significant changes in predation rate following the tarsal exposure. The dorsal application of neonicotinoids to *P. lugubris* (Fig. 1a; one-way ANOVA *F* = 6.3, *D*_*f*_ = 7, *P* < 0.001, power (at α = 0.05) = 1.00) led to a decrease in predation rate. The post-tests revealed that significant effects were observed following the application of imidacloprid at 1183.5 ng cm^−2^ (3.6 ± 0.5 killed *Drosophila melanogaster*; Student-Newman-Keul’s post-test vs. water-treated group *P* < 0.05), whereas the other formulations did not have significant effects on the predation rate (Student-Newman-Keul’s post-tests *P* > 0.05).

The importance of testing multiple species was highlighted by the results obtained using an identical experimental design but involving *P. cespitum* (Fig. 1b). The dorsal application of neonicotinoids to *P. cespitum* decreased the predation rate to all tested neonicotinoid formulations (one-way ANOVA *F* = 5.7, *D*_*f*_ = 7, *P* < 0.001, power (at α = 0.05) = 1.00; Student-Newman-Keul’s post-tests vs. water-treated group *P* < 0.05) except for the low concentration of thiamethoxam (Student-Newman-Keul’s post-test vs. water-treated group *P* > 0.05). Compared with the 5.5 ± 0.5 *D. melanogaster* killed by the control cohort of *P. cespitum*, the decreases in the predation rate of *P. cespitum* were severe, and the numbers of *D. melanogaster* killed reached 2.4 ± 0.4 for thiamethoxam, 2.1 ± 0.4 for thiacloprid, 2.8 ± 0.6 for imidacloprid, and 0.9 ± 0.4 for acetamiprid (data for the high concentration of the two tested concentrations of each insecticide). Concerning tarsal exposure (Fig. 1c), the effects were mild, and thus the experimental settings did not allow to test for differences among them (power (at α = 0.05) = 0.08).

**Figure 2:**
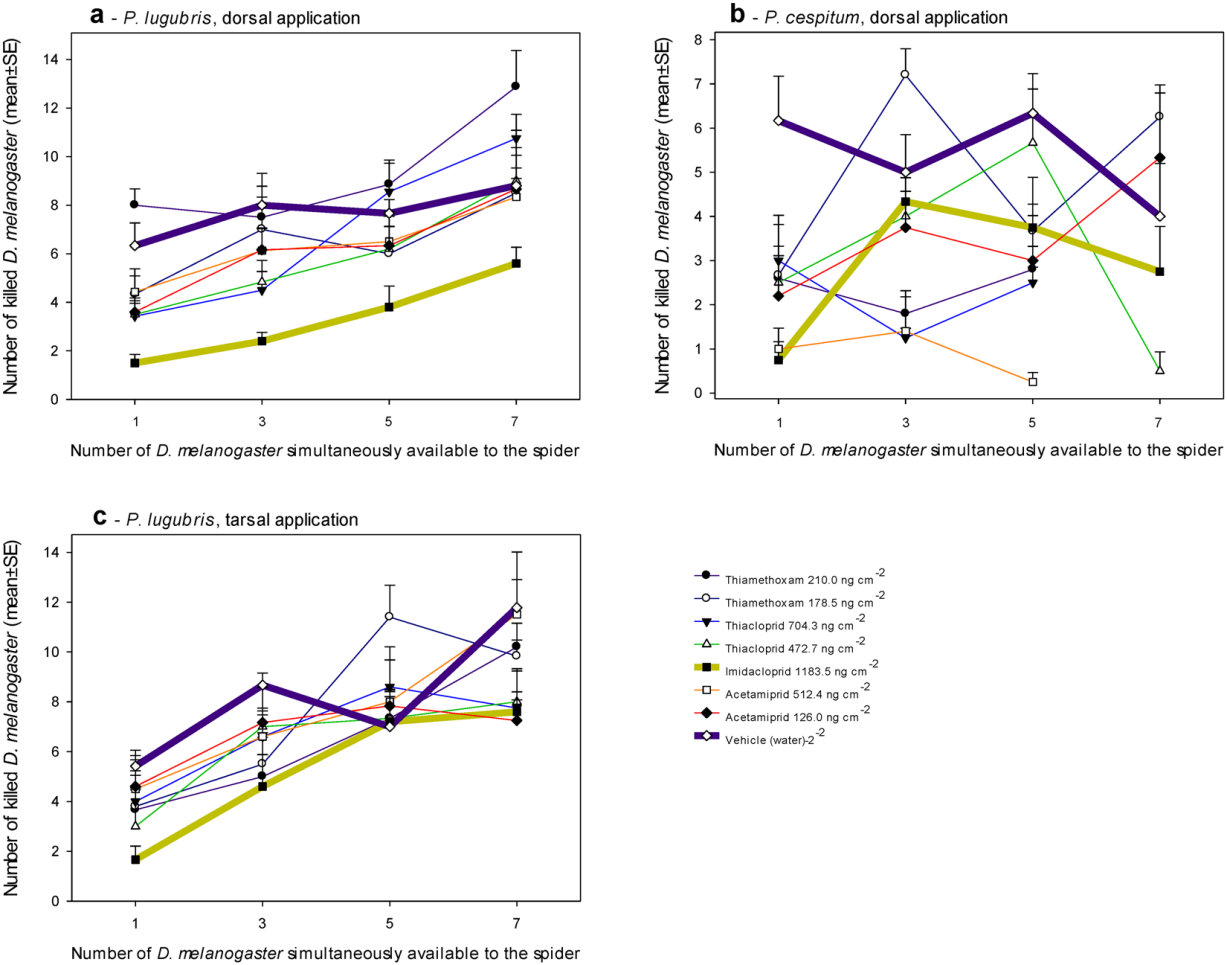
The effects of the simultaneous presence of different numbers of *D. melanogaster* in the test Petri dishes on the predation rate of spiders exposed to the neonicotinoids. Thick lines indicate the controls treated with distilled water only and the cohorts treated with imidacloprid.

The number of *D. melanogaster* simultaneously available to the tested spiders over the course of the experiment affected the predation rate of *P. lugubris* subjected to mock dorsal application (Levene’s equal variance test *P* < 0.05; Kruskal-Wallis one-way ANOVA on ranks *H* = 17.1, *D*_*f*_ = 3, *P* < 0.001) but not that of mock-treated *P. cespitum* (one-way ANOVA *F* = 0.96, *D*_*f*_ = 3, *P* > 0.05) (Fig. 2). Similar trends in density-dependent predation rate of *P. lugubris* were retained in spiders treated dorsally with imidacloprid at 1183.5 ng cm^−2^ (one-way ANOVA *F* = 4.6, *D*_*f*_ = 3, *P* = 0.02, power (at α = 0.05) = 0.66), thiamethoxam at 210.0 ng cm^−2^ (one-way ANOVA *F* = 4.0, *D*_*f*_ = 3, *P* = 0.02, power (at α = 0.05) = 0.61), acetamiprid at 512.4 ng cm^−2^ (not tested because of power (at α = 0.05) = 0.28) and thiacloprid at 704.3 ng cm^−2^ (one-way ANOVA *F* = 9.4, *D*_*f*_ = 3, *P* < 0.001, power (at α = 0.05) = 0.98) (Fig. 2).

### Paralysis and mortality of sheet weavers

The data set of examined Linyphiidae spiders was heterogeneous, represented by 17 species. Therefore, we analyzed separately the dominant species, *Oedothorax apicatus* (Blackwall, 1850), with 331 individuals (72% of the total). The second analyzed group was formed by the other 16 Linyphiidae spp. (Table S1). The second group also included one male and one female of *Collinsia inerrans* (O. P.-Cambridge, 1885) (Araneae: Linyphiidae), which represented the second record of this species for the Czech Republic^39^; the present record comes from a winter wheat field, leg., det. & coll. Milan Řezáč.

**Figure 3:**
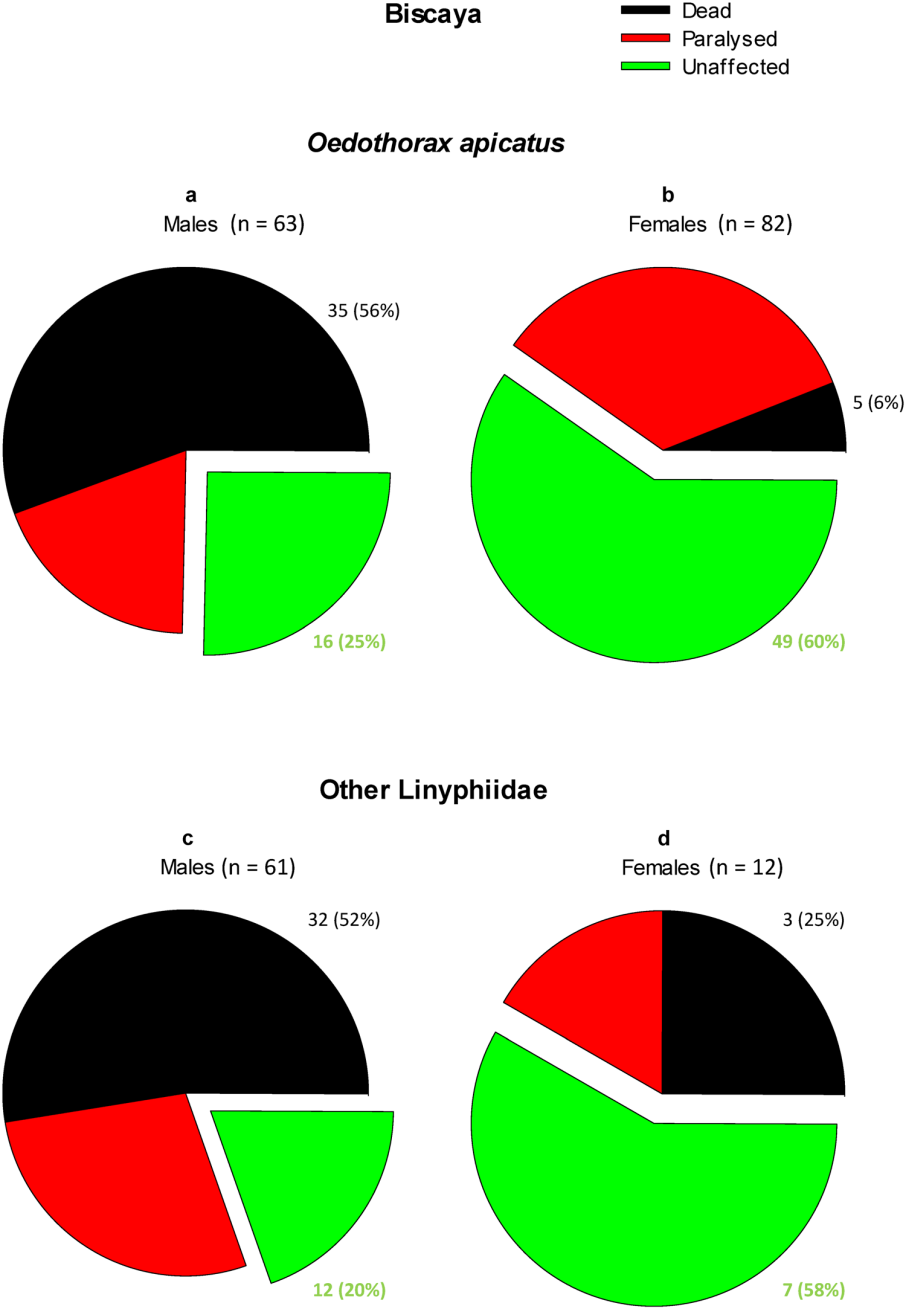
The lethal and sublethal effects of 24-h dorsal treatment of Linyphiidae spp. with Biscaya 240 OD (thiacloprid). (**a**) *O. apicatus* males; (**b**) *O. apicatus* females; (**c**) males of other Linyphiidae spp.; (**d**) females of other Linyphiidae spp.

The dorsal treatment of *O. apicatus* with thiacloprid at 704.3 ng cm^−2^ resulted in sex-specific response of the treated spiders (χ^2^ = 43.918, *D*_*f*_ = 2, *P* < 0.001). The thiacloprid treatment was lethal to 56% of males (Fig. 3a) but only 6% of females (Fig. 3b). By contrast, the portion of paralyzed individuals was similar in both sexes; paralysis affected roughly one-fifth of the examined *O. apicatus*. The results were similar with regards to sex-specific differences when considering the other Linyphiidae spp. (χ^2^ = 7.819, *D*_*f*_ = 2, *P* = 0.02). Despite 52% of males were killed by the thiacloprid treatment at 704.3 ng cm^−2^ (Fig. 3c), the same treatment was lethal to only 25% of the equally treated females (Fig. 3d). The portion of paralyzed individuals was again similar in both sexes. Thus, only 25% of *O. apicatus* males and 20% of the other Linyphiidae males survived a short, one-day-long dorsal contact with thiacloprid without visible, severe health effects.

**Figure 4:**
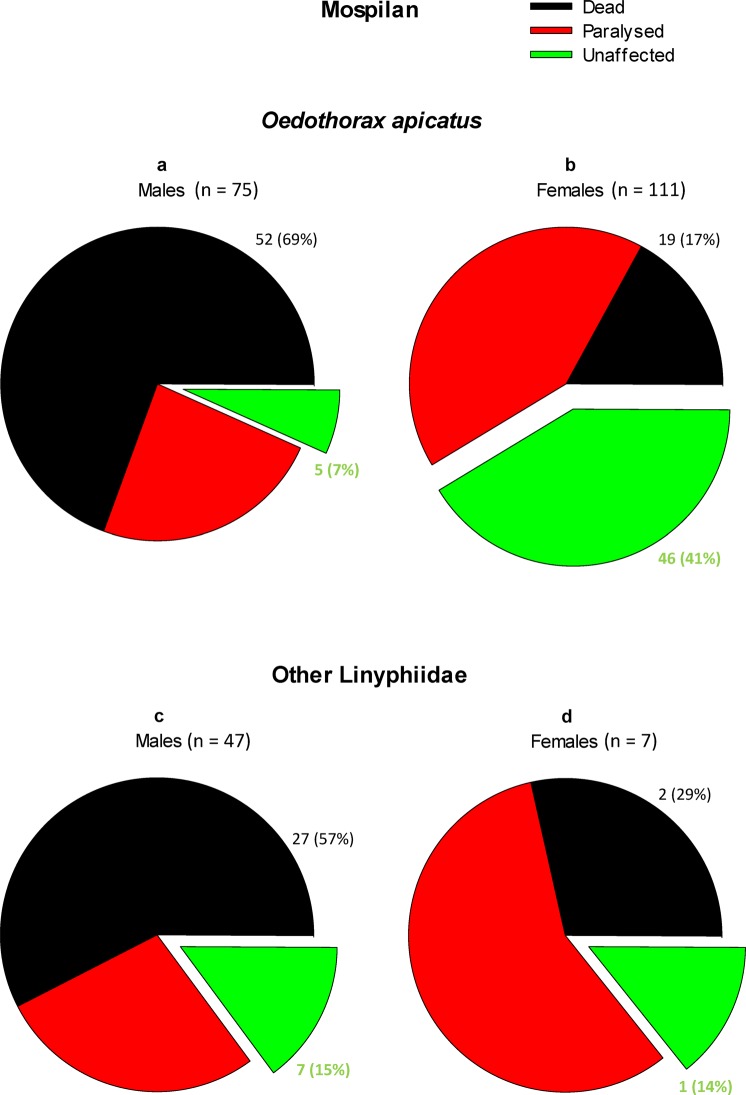
The lethal and sublethal effects of 24-h dorsal treatment of Linyphiidae spp. with Mospilan 20 SP (acetamiprid). (**a**) *O. apicatus* males; (**b**) *O. apicatus* females; (**c**) males of other Linyphiidae spp.; (**d**) females of other Linyphiidae spp.

The dorsal treatment of *O. apicatus* with acetamiprid revealed similar trends as that with thiacloprid. The responses displayed sex-specific differences (χ^2^ = 55.666, *D*_*f*_ = 2, *P* < 0.001). The acetamiprid treatment was lethal to 69% of males (Fig. 4a) but only 17% of females (Fig. 4b). Another 24% of males and 41% of females of *O. apicatus* were paralyzed. Concerning males of the other Linyphiidae spp., the results were similar, with 57% of males killed and 28% of females paralyzed by the acetamiprid treatment (Fig. 4c). The numbers of treated females of the other Linyphiidae spp. were low (see Table S1) but the trends matched the data from other examined groups (Fig. 4d). Thus, only 7% of *O. apicatus* males and 15% of other Linyphiidae males survived a short, one-day-long dorsal contact with acetamiprid without visible, severe health effects.

## Discussion

Although spiders have high functional response, their numerical response is limited due to the low rate of their population growth caused mainly by slow development and limited dispersal^40^. In the present study, we provided the first evidence that the neonicotinoid formulations that are used in agriculture have adverse effects on the predation rate of multiple spider guilds. We showed that the topical application of any of the four tested compounds at recommended field concentrations led to adverse health effects, with imidacloprid associated with the most severe effects on predation rate in different densities of prey. The strong response of spiders to imidacloprid was in line with previous observations, such as the field experiments that were performed in imidacloprid-treated and control wheat fields in Hebei, China, where the spiders and other predators of *Rhopalosiphum padi* and *Sitobion avenae* (Hemiptera: Aphididae) were present at much lower abundance in imidacloprid-treated compared to control sites^41^. However, the other neonicotinoids also caused adverse effects. Particularly when we tested acetamiprid and thiacloprid with a day-long dorsal contact with Linyphiidae spiders, we recorded very high mortality and paralysis of a large part of the surviving Linyphiidae spiders of multiple species (Figs 3 and 4). Additionally, acetamiprid displayed strong sublethal effects (decreased predation rate or paralysis) when applied dorsally to *P. cespitum* (Fig. 1b). Among the four tested neonicotinoids, acetamiprid was previously suggested to be less toxic compared with the others and is subject to less strict regulation in the European Union. However, multiple independent experiments that were performed in the present study did not support this view. The present study compared the effects of application to species with different ecologies and taxonomic classifications. Previously unnoticed were strong differences with respect to species- and sex-specific sensitivities to the study compounds. However, species- or even strain-specific differences in the sensitivity to imidacloprid were previously reported for insects^42^. Such differences prevent straightforward generalization of results obtained from only one or a few common model species and may argue against the claims of limited effects of neonicotinoids on spiders.

In addition to effects on the predation rate in different densities of prey, we found that a day-long topical exposure to neonicotinoids leads frequently to spider paralysis. The observation of paralysis in response to neonicotinoids is actually very common in honey bees, which are often found immobile in front of hives in response to the application of neonicotinoids^43,44^. A recent study reported that the kinetics of paralysis induction is concentration dependent, with concentrations increased by one order of magnitude decreasing the time from exposure to paralysis from 36 hours to only a single hour^45^. The paralytic effects of neonicotinoids are not limited to honey bees. Neurotoxic symptoms, including paralysis, were reported as common also among predatory insects treated with neonicotinoids. For example, for *Harmonia axyridis* (Pallas, 1773) (Coleoptera: Coccinellidae), 72% of larvae treated with thiamethoxam or clothianidin developed neurotoxic symptoms, including paralysis^46^. However, severe toxicity of neonicotinoids is usually limited to species that are not strictly predatory but to those that are rather zoophytophagous^47^ and reports from strictly predatory arthropods, such as spiders, are scarce. The described decrease in predation rate and frequent occurrence of paralysis in the present study suggests that neonicotinoids also cause other neurological defects in spiders, similarly to the spectrum of those known from insects. These effects may change the spectrum of prey the spiders are able to catch. Even the risk of predation by healthy spiders is associated with species-specific escape/defensive behavior^17,48^, which is likely to be more prominent when spiders are intoxicated with neonicotinoids.

The evidence in this paper strongly indicated that neonicotinoid formulations, which are commonly used in agriculture, induced strong lethal and sublethal effects in terms of decreased predation rate and paralysis of spiders. We clearly rejected the null hypothesis claiming that neonicotinoids do not have any effects on the predation rate of central European farmland spiders. The finding of lethal and sublethal effects of neonicotinoids on spiders provides important information for the ongoing debate on the extent of the moratorium on the use of neonicotinoids in agriculture and supports the position of the European Union that banned the use of imidacloprid, clothianidin and thiamethoxam in the field although they are still allowed for use in glasshouses^22^. The effects of chronic exposure to low concentrations of these compounds and the possible existence of thresholds for acute intoxication remain to be investigated. Moreover, the present experimental design consisted of topical application only. When comparing topical and oral applications in arthropods, the concentrations or doses that kill 50% of individuals are usually lower by one order of magnitude for the oral applications compared to the topical ones^49^. The data provided in the present study call for attention to the role of neonicotinoids in shaping farmland arthropod communities beyond insects and mites.

## Material and Methods

### Predation rate in different densities of prey of epigeic and epiphytic spiders

We used an experimental model that involved epigeic (*P. lugubris*) and epiphytic (*P. cespitum*) species of spiders, which are both known as dominant spider species in central European orchards^50,51^. We collected subadult or adult females of the two species several days before the experiments in the Prague-Ruzyně (50.09°N, 14.30°E, 349 m a.s.l.) environment and acclimated them at 22 °C and 80% humidity and under a natural day/night regimen. In total, we examined 782 *P. lugubris* and 158 *P. cespitum*. Both species actively search for prey on the ground or plant surfaces. For spider prey, we used cultures of wingless *D. melanogaster* cultivated on nutrient-rich medium.

We tested four neonicotinoid insecticides in formulations that are commonly used to spray crops to eliminate pest insects, and which are listed in Table 1. We excluded the neonicotinoids that are used only for the treatment of seeds before they are sown (for example, clothianidin). We applied the neonicotinoids at concentrations recommended by the manufacturers for spray applications under field conditions (Table 1). In addition to exposure via contaminated food resources, two routes of topical exposure to neonicotinoids occur in nature: direct contact with spray droplets and contact with residues on sprayed surfaces. In the present study, we mimicked topical contact by a) direct spraying of the dorsal side of the body and b) application to the leg tarsi, which contact contaminated surfaces during walking. For the dorsal application, we placed spiders of both tested species in 24-well plates and sprayed them with a controlled amount of the neonicotinoids in distilled water, or distilled water alone, using the Potter Precision Laboratory Spray Tower (Burkard Scientific, Uxbridge, UK). For the tarsal exposure, we sprayed empty wells with neonicotinoids at concentrations identical to those in the previous experiment. The droplets were allowed to dry, and then *P. lugubris* spiders were introduced and allowed to have primarily tarsal contact with neonicotinoid residues. In both experiments, spider mortality was recorded after one hour of exposure. The spiders that did not die during the initial one-hour-long period were later tested for predation rate in different densities of prey (intake rate of a spider as a function of food density). Because of the excluded individuals, the sizes of tested groups were not mutually identical, and reached 18–22 individuals per formulation for tarsal exposures of *P. lugubris* (with n = 45 for the mock-treated group), 17–32 individuals per formulation for dorsal applications to *P. lugubris*, and 9–22 individuals per formulation for dorsal applications to *P. cespitum*. To perform these tests, the spiders were placed individually in 40 mm Petri dishes, and living wingless *D. melanogaster* were provided to the spiders for ten hours. The dishes were checked every 15 min, and any killed flies were replaced with living ones to maintain a constant prey density for the tested spiders. After ten hours, the killed flies were counted. The effect of the simultaneous presence of different numbers of flies (1, 3, 5 or 7 flies present simultaneously) in the experimental Petri dishes was tested under the above-described experimental settings.

**Table 1:** Formulations of neonicotinoid insecticides that we used in the present study.

Insecticide	Formulation	Manufacturer	Active substance content [%]	Recommended application rate	Recommended concentration [ng cm^−2^]
Thiamethoxam	Actara 25 WG	Syngenta Crop Protection, Basel, Switzerland	25	70–80 mg ha^−1^	178.5–210.0
Thiacloprid	Biscaya 240 OD	Bayer CropScience, Monheim am Rhein, Germany	27.97	200–300 ml ha^−1^	472.7–704.3
Imidacloprid	Confidor 200 OD	Bayer CropScience, Monheim am Rhein, Germany	19.3	600 ml ha^−1^	1183.5
Acetamiprid	Mospilan 20 SP	Nippon Soda Co., Tokyo, Japan	20	60–250 ml ha^−1^	126.0–512.4

### Paralysis of sheet weavers

To corroborate the observed effects on another group of spiders, we collected spiders of the family Linyphiidae using dry pitfall traps that were deployed for a single day in the winter wheat field near Lány (50.13°N, 13.96°E, 427 m a.s.l.) environment from June 20 to June 23, 2018. For the purpose of the experiments, we nonselectively collected all captured adult Linyphiidae spiders, placed them individually into wells of flat-bottom polystyrene microplates and acclimated them at 22 °C and 80% humidity and under a natural day/night regimen. We applied dorsally the same concentrations of neonicotinoids as in the predation rate in different densities of prey experiments, namely Biscaya (704.3 ng of thiacloprid cm^−2^) and Mospilan (512.4 ng of acetamiprid cm^−2^), and checked the conditions of the spiders (alive, paralyzed or dead) 24 hours after the treatment. Because the identification of Linyphiidae spiders often requires a check of genitalia, we identified them to species only after the experiments were terminated. We identified the collected spiders based on Nentwig *et al*.^52^ using the nomenclature based on the World Spider Catalog^53^.

### Statistical analyses

Each spider was treated individually, considered a single experimental unit. Data are shown as the mean ± SE unless stated otherwise. We used one-way ANOVA to test for differences between treatments and control, which was followed by Student-Newman-Keul’s post-tests. We used the Shapiro-Wilk normality test and Levene’s equal variance test to analyze the distribution of the data. In the case any of these tests revealed significant deviations from normality or equal variance, we employed Kruskal-Wallis one-way ANOVA on ranks instead of the one-way ANOVA. We used Fisher’s exact tests to analyze the survival data with a total N ≤ 100, and the χ^2^ test with Yates correction for continuity to analyze the survival data with a total N > 100 and the sex-specific differences in the numbers of paralyzed and dead Linyphiidae spp. Because previously obtained data on the effects of neonicotinoids on the predation rate in different densities of prey of spiders were not available, we chose the size of the tested cohorts ad hoc and performed a post-test power analysis on the obtained data. The analyses were conducted in SigmaPlot 12.0.

## Supplementary information

Table S1

## Data Availability

All data generated or analysed during this study are included in this published article (and its Supplementary Information files).
