# Supplementary material for: Contact application of neonicotinoids suppresses the predation rate in different densities of prey and induces paralysis of common farmland spiders
Source: Sci Rep. 2019 Apr 5;9:5724. doi: 10.1038/s41598-019-42258-y (PMC6450932; doi:10.1038/s41598-019-42258-y)
Supplement: Supplementary file 1 — Table S1 [file 41598_2019_42258_MOESM1_ESM.pdf]

Supplementary Materials for

Contact application of neonicotinoids suppresses the predation rate in different densities of prey and induces paralysis of common farmland spiders

Milan Řezáč, Veronika Řezáčová, Petr Heneberg

This file includes:

Table S1

Table S1. List of Linyphiidae spp. used in the experiments with contact application of acetamiprid and thiacloprid. The table contains the complete list of Linyphiidae spp. found. For the purpose of analyses, these species were split to *Oedothorax apicatus* and all other Linyphiidae spp. Indicated are the numbers of males and females found and used, assignment of the individuals to the experiments and raw data that resulted from the experiments.

	Total				Thiacloprid			Acetamiprid		
	Males	Females	All sexes		Healthy	Paralysed	Dead	Healthy	Paralysed	Dead
Oedothorax apicatus (Blackwall, 1850)	138	193	331	Males	16	12	35	5	18	52
				Females	49	28	5	46	46	19
Micrargus subaequalis (Westring, 1851)	16	0	16	Males	1	4	9		1	1
				Females						
Erigone atra Blackwall, 1833	26	2	28	Males	5	5	5	2	1	8
				Females	1					1
Erigone dentipalpis (Wider, 1834)	22	2	24	Males	2	4	7		4	5
				Females	1	1				
Agyneta rurestris (C. L. Koch, 1836)	27	3	30	Males	3	1	4	3	7	9
				Females	2		1			
Walckenaeria atrotibialis (O. P.-Cambridge	4	1	5	Males	1	1	2			
				Females					1	
Diplostyla concolor (Wider, 1834)	1	3	4	Males						1
				Females	1		1		1	
Mermessus trilobatus (Emerton, 1882)	1	1	2	Males			1			
				Females	1					
Walckenaeria vigilax (Blackwall, 1853)	1	1	2	Males		1				
				Females					1	
Pocadicnemis pumila (Blackwall, 1841)	0	1	1	Males						
				Females		1				
Porrhomma microphthalmum (O. P.-Camb	3	2	5	Males		1	1	1		
				Females			1			1
Oedothorax fuscus (Blackwall, 1834)	1	0	1	Males						1
				Females	1					
Collinsia inerrans (O. P.-Cambridge, 1885)	1	1	2	Males						1
				Females				1		
Diplocephalus cristatus (Blackwall, 1833)	1	0	1	Males						1
				Females						
Tenuiphantes tenuis (Blackwall, 1852)	1	0	1	Males				1		
				Females						
Diplocephalus latifrons (O. P.-Cambridge, 1	2	1	3	Males			2			
				Females					1	
Araeoncus humilis (Blackwall, 1841)	1	0	1	Males			1			
				Females						
